# Variable ecological conditions promote male helping by changing banded mongoose group composition

**DOI:** 10.1093/beheco/arw006

**Published:** 2016-01-26

**Authors:** Harry H. Marshall, Jennifer L. Sanderson, Francis Mwanghuya, Robert Businge, Solomon Kyabulima, Michelle C. Hares, Emma Inzani, Gladys Kalema-Zikusoka, Kenneth Mwesige, Faye J. Thompson, Emma I. K. Vitikainen, Michael A. Cant

**Affiliations:** 1 ^a^ Centre for Ecology and Conservation, University of Exeter, Cornwall Campus, Treliever Road, Penryn, Cornwall TR10 9FE, UK,; 2 ^b^ Banded Mongoose Research Project, Queen Elizabeth National Park, Kasese, Uganda,; 3 ^c^ Conservation Through Public Health, Plot 3, Mapeera Lane, Entebbe, Uganda

**Keywords:** cooperation, ecological variability, environmental change, individual differences, sex ratios.

## Abstract

Ecological conditions are expected to have an important influence on individuals’ investment in cooperative care. However, the nature of their effects is unclear: both favorable and unfavorable conditions have been found to promote helping behavior. Recent studies provide a possible explanation for these conflicting results by suggesting that increased ecological variability, rather than changes in mean conditions, promote cooperative care. However, no study has tested whether increased ecological variability promotes individual-level helping behavior or the mechanisms involved. We test this hypothesis in a long-term study population of the cooperatively breeding banded mongoose, *Mungos mungo*, using 14 years of behavioral and meteorological data to explore how the mean and variability of ecological conditions influence individual behavior, body condition, and survival. Female body condition was more sensitive to changes in rainfall leading to poorer female survival and pronounced male-biased group compositions after periods of high rainfall variability. After such periods, older males invested more in helping behavior, potentially because they had fewer mating opportunities. These results provide the first empirical evidence for increased individual helping effort in more variable ecological conditions and suggest this arises because of individual differences in the effect of ecological conditions on body condition and survival, and the knock-on effect on social group composition. Individual differences in sensitivity to environmental variability, and the impacts this has on the internal structure and composition of animal groups, can exert a strong influence on the evolution and maintenance of social behaviors, such as cooperative care.

## Lay Summary

Male banded mongooses babysit more when rainfall is variable. Banded mongooses live in cooperative family groups and males in particular help raise pups that are not necessarily their own. It has been suggested that ecological conditions affect cooperation, and our study confirms that the variability of conditions is important: Females face higher mortality during years with more variable rainfall, and males may be better off helping their relatives when there are fewer opportunities for mating.

Twitter: @HarryHMarshall

## INTRODUCTION

In cooperatively breeding groups, individuals can gain direct fitness by competing with other group members for reproductive opportunities or gain indirect fitness by helping to care for relatives’ offspring. Ecological conditions are expected to influence individuals’ investment in helping because these conditions can constrain or promote their ability to reproduce independently (Emlen 1982; Hatchwell and Komdeur 2000; McLeod and Wild 2013). According to Hamilton’s (1964) rule, selection will favor helping behavior when *br* > *c*, that is, when the fitness benefits to others (*b*), weighted by the actor’s relatedness to them (*r*), outweigh the fitness costs incurred by the actor (*c*). Much research has focused on the influence of relatedness on selection for helping behavior (Abbot et al. 2011; Liao et al. 2015). However, the 2 terms of Hamilton’s rule that are most sensitive to ecological conditions, the fitness costs and benefits (*c* and *b*), have received less attention, and the precise role of ecological variation in the evolution of cooperative breeding is a matter of debate (Foster and Xavier 2007; Gardner and Foster 2008; Cockburn and Russell 2011).

Previous tests of the impact of ecological conditions on helping behavior have focused on the effect of changes in mean ecological conditions and have yielded conflicting results. Individuals tend to increase their investment in helping behavior when opportunities to occupy independent breeding positions are limited, but this can occur both where conditions are favorable and all positions are occupied, that is, habitats are saturated (Komdeur 1992; Schoepf and Schradin 2012), or where adverse conditions limit the total number of these positions (Russell 2001; MacColl and Hatchwell 2002; Hatchwell and Sharp 2013). Ecological conditions can also promote helping by increasing the net fitness benefits to individuals that help, for example, through reduced aggression from group members, but again this has been shown in both favorable (Dickinson and McGowan 2005; Baglione et al. 2006; Koenig et al. 2011) and adverse conditions (Shen et al. 2012).

One reason for the lack of consensus across these studies may be that they do not consider the effect of the variability in ecological conditions. Recent comparative studies have shown that cooperatively breeding bird species are more likely to occur in variable environments (Rubenstein and Lovette 2007; Jetz and Rubenstein 2011; but see Gonzalez et al. 2013), suggesting that ecological variability might promote helping behavior. But what mechanism could generate this relationship? One explanation is supplied by theoretical work showing that high variance in fecundity selects for helping behavior (Lehmann and Balloux 2007). This work is rooted in the principle established by Gillespie (1974, 1975, 1977) that selection against variance in fecundity is stronger in small populations, and more recent work showing that the same selection against variance in fecundity occurs when populations are structured into local breeding groups with limited dispersal (Shpak and Proulx 2007), as is often the case in cooperative breeders. Informed by this theoretical work, Rubenstein (2011) hypothesized that helping behavior in cooperative breeders is a bet-hedging strategy adopted by subordinate individuals in more variable ecological conditions, allowing them to reduce their variance in fecundity when the variance associated with independent breeding is high. In support of this hypothesis, he showed that variance in superb starling (*Lamprotornis superbus*) groups’ reproductive success was higher in periods associated with more variable rainfall, and lower when groups (and so the number of helpers) were larger. However, neither Rubenstein nor any other study to our knowledge has tested the effect of ecological variability on individual-level helping decisions, and so the prediction that subordinates should increase their helping effort in variable environments.

An alternative to Rubenstein’s hypothesis is that variability promotes helping by changing the social environment in which helping occurs. Cooperative groups consist of individuals who differ in numerous ways that may affect their response to changes in ecological conditions. In particular, there is considerable empirical evidence that sex differences in mortality can occur due to differences in sensitivity to ecological conditions (Conradt et al. 2000; Coulson et al. 2001), and so it seems plausible that ecological variability might affect the survival and reproduction of males and females differently. Such sex differences in mortality may also have important impacts on selection for social traits. For example, Mullon et al. (2014) showed that sex differences in reproductive variance are predicted to influence the evolution of behaviors such as parental care and that the relative strength of selection for maternal versus paternal care depends on sex differences in mortality. Helping effort can also vary with sex (Clutton-Brock et al. 2002; Hodge 2007; Bell 2010), and males and females have been shown to respond differently in how they alter their helping effort in response to changes in individual condition and environmental quality (Hodge 2007; Nichols, Amos, et al. 2012). Finally, sex differences in mortality are likely to affect group sex ratios and so the level of reproductive competition, which has itself been shown to influence individual helping and reproductive effort (Kvarnemo et al. 1996; Clutton-Brock et al. 2006; Clutton-Brock and Huchard 2013). Consequently, sex differences in susceptibility to ecological variability, and the knock-on effects on group composition, provide an alternative hypothesis to explain why ecological variability might influence helping behavior.

In this study, we tested these hypotheses using a tractable wild mammal system, the banded mongoose (*Mungos mungo*). We used a detailed 14-year data set describing the mean and variability of rainfall in the previous 12 months (as a proxy for ecological conditions) and mongoose helping effort, body condition, and survival to address 4 questions. 1) How does the mean and variability of rainfall influence individuals’ reproductive and helping effort, and do these effects depend on individuals’ age and sex? Banded mongoose groups contain age-ranked dominance hierarchies (Cant et al. 2013) and so Rubenstein’s (2011) hypothesis predicts that younger (subordinate) individuals should increase helping effort in more variable ecological conditions. However, as we show below, our results suggest that only older (dominant) males show such an increase. Consequently, we explore the alternative hypothesis that sex differences in the impacts of ecological variability explain variation in helping effort. Specifically, we ask 2) whether the effect of rainfall on individual body condition and survival differs between the sexes and 3) whether ecological impacts on group composition—in particular, the number of adults (potential helpers) and adult sex ratio—can explain observed patterns of helping and reproductive behavior. Finally, we tested 4) the assumption that increases in reproductive and helping effort have positive impacts on direct and indirect fitness, respectively.

## METHODS

### Study system

We conducted our study between October 1999 and June 2013 on the Mweya Peninsula in Queen Elizabeth National Park, Uganda (0°12′S, 27°54′E). The banded mongooses at this site are part of a long-term study population, and we provide details here about banded mongoose biology and our study site specific to this study; an in-depth description of both can be found in Cant et al. (2013) and references therein.

Banded mongooses are small (<2kg) diurnal herpestids that live in stable multimale, multifemale groups of typically between 10 and 30 individuals. All adult females enter estrus within days of each other, and males compete for mating opportunities by guarding females and physically blocking other males’ access. Females usually give birth synchronously on the same morning to large litters around 4 times a year. Pups stay in an underground den for approximately 30 days and are guarded during the day by one or more “babysitters” while the rest of the group forages elsewhere. After emerging from the den, pups forage with the group and are cared for by adult “escorts” for a further 30 days. All mongooses in the study population are individually marked using either unique hair-shave patterns or color-coded collars and are habituated to close observation from at least 5 m. One to 2 mongooses in each group are fitted with a radio collar weighing 26–30g (Sirtrack Ltd, Havelock North, New Zealand) with 20-cm whip antenna (Biotrack Ltd, Dorset, UK) to allow the groups to be located. Two groups have access to human refuse (Otali and Gilchrist 2004) and so were excluded from this study.

### Data collection

We collected climate data daily from a weather station situated centrally at our study site. We selected rainfall as our proxy of ecological conditions because it is relatively variable at Mweya (mean monthly rainfall ± standard deviation [SD] = 61±41mm, *n* = 152 months), whereas temperature is reasonably constant (mean of monthly mean maximum daily temperature ± SD = 29.5±1.5 °C, *n* = 162 months). In addition, increased rainfall has been associated with vegetation change and increased invertebrate abundance at Mweya (Rood 1975; Lock 1993; Cant et al. 2013) and increases in mongoose helping and reproduction (Nichols, Amos, et al. 2012; Nichols, Bell, et al. 2012), showing that rainfall is a relevant measure of ecological conditions the mongooses are experiencing. The annual rainfall cycle at Mweya involves 2 dry seasons from January to February and from June to July dividing 2 distinctly different wet seasons: a short wet season from March to May and a long wet season from August to December. The short season is more intense (mean ± SD monthly rainfall: short season = 94±47mm, *n* = 36 months; long season = 73±33mm, *n* = 60 months), but the long season is wetter overall (mean total rainfall: short season = 281±49mm, *n* = 12 years; long season = 366 m ± 46mm, *n* = 12 years). Therefore, to capture changes in the mean and variability of ecological conditions across this cycle, we used the mean and SD of the monthly rainfall in the previous 12 months as our proxies of ecological conditions. Our use of these year-long measures of rainfall is consistent with previous studies of the ecological variability and cooperative breeding, which have also characterized the environment on the basis of the mean of and variation in annual climatic measures (Rubenstein and Lovette 2007; Jetz and Rubenstein 2011; Rubenstein 2011; Gonzalez et al. 2013).

We visited banded mongoose study groups every 1–3 days. Each visit lasted at least 20min during which the presence or absence of individuals was noted. We were able to differentiate between absence due to death and absence due to dispersal because although death involves the permanent disappearance of single individuals, dispersal in banded mongooses occurs through the eviction of multiple individuals from the group at the same time and is often preceded by a conspicuous period of aggression between individuals in the group (Cant et al. 2001, 2010). When study groups had pups in their den, we measured individuals’ investment in helping as the proportion of group visits in a breeding attempt that they were at the den babysitting (and so incurring a cost by not being able to forage and interact with the rest of the group). We identified babysitting individuals in 2 ways: 1) by directly observing them at the den while the rest of the group was away (e.g., if the babysitter was wearing a radio collar) and 2) by their temporary absence from the group while it was away from the den, with the individual being observed with the group at the next visit. When the group’s females were in estrus, we measured males’ investment in reproduction as the proportion of group visits they were observed mate-guarding a female. Males were defined as mate-guarding when they were closely (<30cm) following and blocking other males’ access to estrous females (Cant 2000). Female pregnancy was identified by visual swelling of the abdomen and confirmed by palpation during trapping. Most individuals are trained to step onto portable weighing scales in return for a small milk reward and were weighed weekly in the morning before foraging started and again in the evening once the day’s foraging had finished. When individuals were first trapped, a 2-mm skin sample was taken. DNA was extracted from this and used to assigning parentage using a panel of 43 polymorphic microsatellite markers. See Hodge (2007) and Jordan et al. (2010) for further details of the trapping procedure and Sanderson, Wang, et al. (2015) for details of the parentage assignment.

### Analyses

Our analyses addressed our 4 questions by testing how ecological conditions affected 1) individuals’ reproductive and helping effort, 2) their condition and survival, and 3) the number of adults and the adult sex ratio within their social group. It also tested 4) whether the variation in reproductive and helping effort we found in our analyses had direct and indirect fitness benefits, respectively (as found previously: Cant 2003; Sanderson, Wang, et al. 2015). We conducted our analyses using generalized linear mixed effect models (GLMMs) and Cox proportional hazard models. We fitted our models to data from individuals who were between 2 and 8 years to remove any development and senescence effects, respectively. Separate models were fitted to data from males and females in our helping effort and survival analyses to avoid overparameterization issues, and in our reproductive effort analyses due to its sex-specific nature (mate-guarding by males and estrous/pregnancy state in females). We present the parameter estimates and standard errors (SE) from our full models, rather than reduce our model further due to issues with stepwise model reduction techniques such as erroneously rejecting null hypotheses (type I errors) (Whittingham et al. 2006; Mundry and Nunn 2009; Forstmeier and Schielzeth 2011). We did, however, remove nonsignificant interactions from our final model to allow the significance of main effects not involved in interactions to be tested (Engqvist 2005). We assessed the significance of effects by comparing a model without a particular effect to a full model using likelihood ratio tests (Bates et al. 2015). Correlations between variables fitted in models as fixed effects were lower than the levels shown by Freckleton (2011) to cause model fitting issues such as variance inflation in effect estimates (max *r* = 0.38). In addition to being detailed below, the variables fitted in each model are listed in Supplementary Tables S1–S4. We performed all analyses in R (R Core Team 2014), fitting GLMMs using the lme4 package (Bates et al. 2014) and our Cox proportional hazard models using the survival package (Therneau 2014).

#### Individual reproductive and helping effort

We measured male investment in reproduction as the proportion of group visits during an estrous period they were observed mate-guarding a female. We measured female investment in reproduction as 1) whether they were observed in estrus during a breeding attempt and 2) whether they got pregnant during a breeding attempt. We used 2 measures of female reproductive investment as although female estrous state is probably a more direct measure of her “decision” as to whether to invest in reproduction at a given time, detection of this in banded mongooses is dependent on the presence of a male mate-guard. Therefore, to confirm that our analysis of female estrous state was not biased by male mate-choice behavior, we also analyzed whether a female got pregnant in a breeding attempt as a second measure of reproductive investment. Although this is a less direct measure (because it can be influenced by other factors, e.g., successful conception after mating), it is detected by observation and palpation (see Data collection for details) and so is independent of male behavior. Our reproductive investment models included the following fixed effects: mean and SD in monthly rainfall in the previous 12 months, mean and SD in monthly rainfall in the mongooses’ first year of life, individuals’ age, and the interaction between age and the rainfall measures. Rainfall measures from the mongooses’ first year were included here and in further models to control for early-life effects (Monaghan 2008; English et al. 2013) and are explored elsewhere (Marshall HH et al., unpublished data). Our reproductive investment models also included individuals’ weight at the start of the estrous period, whether the estrous period overlapped with a babysitting or pup-escorting period and the ratio of males to females in the group (mate-guarding model) or total number females in the group (estrous and pregnancy models). This latter set of variables was included to control for, respectively, short-term differences in individual condition (Hodge 2007), conflict with helping demands, and competition from other males or females. The models also included mongoose, breeding attempt, and social group identities as random intercepts and were fitted, respectively, to 335 mate-guarding measures from 68 males in 5 social groups and 184 estrous and pregnancy records from 49 females in 6 social groups using a binomial error structure with logit link function.

We measured individual investment in helping as the proportion of group visits during a breeding attempt that individuals were recorded babysitting. Our models included the following fixed effects: mean and SD in monthly rainfall in the previous 12 months, mean and SD in monthly rainfall in the mongooses’ first year of life, individuals’ age, and the interaction between age and the rainfall measures. They also included individuals’ weight at the start of the babysitting period, whether the babysitting period overlapped with an estrous period and the total number of adults in the group as fixed effects. In a similar fashion to our reproductive investment models, these effects were included to control for short-term changes in body condition, conflict with reproduction, and the number of other potential babysitters. These models also included mongoose, breeding attempt, and social group identities as random intercepts were fitted to 451 and 298 babysitting measures from 78 males in 5 social groups and 55 females in 7 social groups, respectively, using a binomial error structure with logit link function.

#### Condition and survival

We measured changes in mongoose body condition as changes in individuals’ weight on both a daily and annual basis, excluding weights recorded from pregnant females. Models predicting both of these weight change measures included mongoose age, sex, and weight at the start of the day or year as fixed effects, and mongoose and social group identities as random intercepts. The model of daily weight change included rainfall in the previous 30 days and its interaction with age and sex, and was fitted to 3524 daily weight changes recorded from 139 individuals in 10 social groups. The model of annual weight change included the mean and SD in monthly rainfall in the previous 12 months, the mean and SD in monthly rainfall in the mongooses’ first year of life, and these rainfall measures’ interaction with age and sex. It was fitted to 247 annual weight changes (change in morning weight over 365±30 days) recorded from 121 individuals in 8 social groups. Both weight change models were fitted using a normal error structure, and their residuals were checked to ensure they were normally distributed with a constant variance.

We then tested the effect of individual weight changes and ecological conditions on survival. To do this, we fitted a Cox proportional hazard model to a left-truncated, right-censored data set describing whether each individual survived a given year of their life, the mean and SD of the monthly rainfall during that year, and their rate of proportional weight change during the year. We selected start and end weights as the closest weight available to the start or end of observation period ± 30 days, and so we used a rate of proportional weight change to account for differences in the number of days each weight change was measured over. To calculate an individual’s rate of proportional weight change, we calculated their proportional weight change (absolute weight change divided by their weight at the start of the year) and divided it by the number of days between start and end weight. Our survival models included the following fixed effects: rate of proportional weight change, mean and SD of monthly rainfall during the year, the interaction between weight change and these rainfall measures, and the mean and SD of monthly rainfall in the mongooses’ first year of life. They also included individuals’ social group ID as a frailty term; were fitted to data from 75 males in 6 social groups and 39 females in 7 social groups; and their residuals were visually checked for nonproportional hazards, influential observations, and nonlinearities.

#### Social group composition

We tested the effects of ecological conditions on the number of adults (potential helpers) and the proportion of adult males in the group at the start of each breeding attempt. These models included the mean and SD in monthly rainfall in the previous 12 months and rainfall in the past 30 days as fixed effects, and social group identity as a random intercept. They were fitted to adult number and sex ratio measures from 196 breeding attempts from 14 social groups using a binomial error structure and a logit link function.

#### Fitness benefits of reproductive and helping effort

We measured the direct fitness benefit of mate-guarding by fitting a model predicting the probability that a male successfully sired a pup in a breeding attempt (parentage assigned by DNA analysis, see above and Sanderson, Wang, et al. 2015). This model included males’ age and their mate-guarding effort (proportion of visits they were observed mate-guarding) as fixed effects in 2-way interactions with the mean and SD of monthly rainfall in the previous 12 months and rainfall in the past 30 days. The model also included the ratio of males to females in the group as a fixed effect and mongoose ID, breeding attempt, and social group identities as random intercepts and was fitted to 596 records of siring success from 104 males and 98 breeding attempts in 7 social groups using a binomial error structure with a logit link function.

We measured the indirect fitness benefits of babysitting using pup survival, as the probability that a litter emerged from the den after the babysitting period. This model included the following fixed effects: the group babysitting effort (mean number of babysitters recorded per visit) in 2-way interactions with the mean and SD of monthly rainfall in the previous 12 months, rainfall in the past 30 days, the number of females who gave birth in the breeding attempt, and the number of adults (potential helpers) in the group. Social group identity was included as a random intercept, and the model was fitted to emergence records from 142 breeding attempts from 12 social groups.

## RESULTS

### Individual reproductive and helping effort

**Figure 1:**
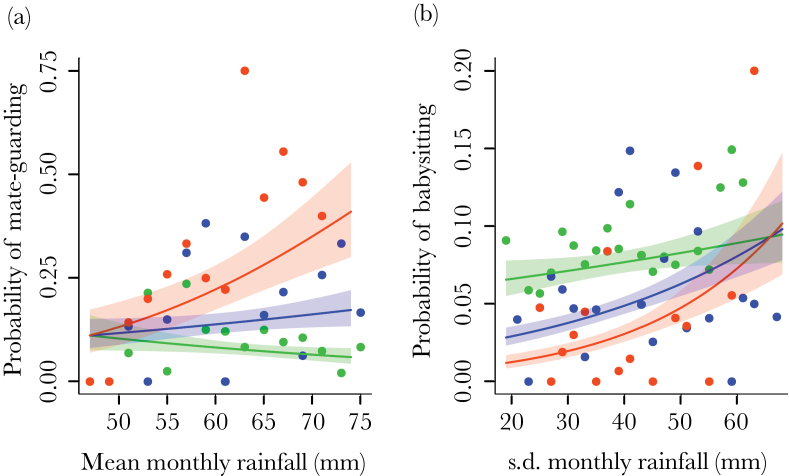
The effect of (a) the mean monthly rainfall in previous 12 months on the probability of a male mate-guarding during a breeding attempt and (b) the SD in monthly rainfall in previous 12 months on the probability of a male babysitting during a breeding attempt. The points are mean values for 2-mm rainfall bins for males aged 2–4 (green), 4–6 (blue), and 6–8 years (orange). The lines are relationships predicted by the models for males aged 3 (green), 5 (blue), and 7 years (orange) with all other effects in the model set at their mean. Shaded areas show the SE of these predictions. Age is categorized here for illustrative purposes and was a continuous variable in our analyses.

Male investment in reproduction and helping was influenced by ecological conditions in differing ways. Males’ mate-guarding was influenced by the mean monthly rainfall in an age-dependent manner (age × mean rainfall: β ± SE = 0.02±0.01, $\chi_{1}^{2}$ = 5.99, *P* = 0.01) but was not affected by its variability ($\chi_{1}^{2}$ = 0.66, *P* = 0.41). Older males were more likely to mate-guard when the mean monthly rainfall in the previous 12 months had been high (Figure 1a). Male babysitting, however, was not affected by the mean monthly rainfall ($\chi_{1}^{2}$ = 0.97, *P* = 0.32) but was affected by its variability, again in an age-dependent manner (age × SD rainfall: β ± SE = 0.01±0.004, $\chi_{1}^{2}$ = 5.34, *P* = 0.02). Specifically, older males invested more in babysitting when rainfall in the past year had been more variable (Figure 1b). This pattern is the opposite to Rubenstein’s (2011) prediction that helping effort in subordinates (younger individuals here) should increase with ecological variability. In contrast to males, female investment in reproduction and helping was not affected by ecological conditions. Changes in the mean and variability of rainfall did not influence the probability of females being in estrus (mean rainfall: $\chi_{1}^{2}$ = 2.16, *P* = 0.14; SD rainfall: $\chi_{1}^{2}$ = 0.06, *P* = 0.80), getting pregnant (mean rainfall: $\chi_{1}^{2}$ = 1.97, *P* = 0.16; SD rainfall: $\chi_{1}^{2}$ = 0.10, *P* = 0.76), or babysitting (mean rainfall: $\chi_{1}^{2}$ = 2.53, *P* = 0.11; SD rainfall: $\chi_{1}^{2}$ = 0.11, *P* = 0.74; Supplementary Table S1).

### Condition and survival

**Figure 2:**
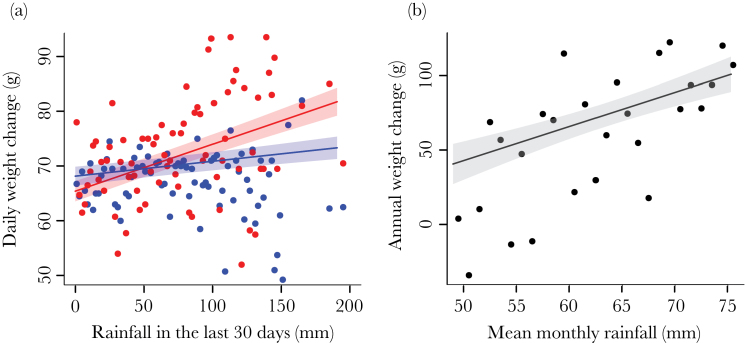
Rainfall and individual weight gain: the effect of (a) rainfall in the past 30 days on daily weight gain and (b) mean monthly rainfall in the past 12 months on the annual weight gained by mongooses. In panel (a), red signifies females and blue signifies males. Points show the mean weight gains in (a) 2-mm bins up to 160 mm and then 10-mm bins from 160 to 200 mm, and (b) 2-mm bins. In both panels, the lines and shaded areas are the model predictions ± SE.

**Figure 3:**
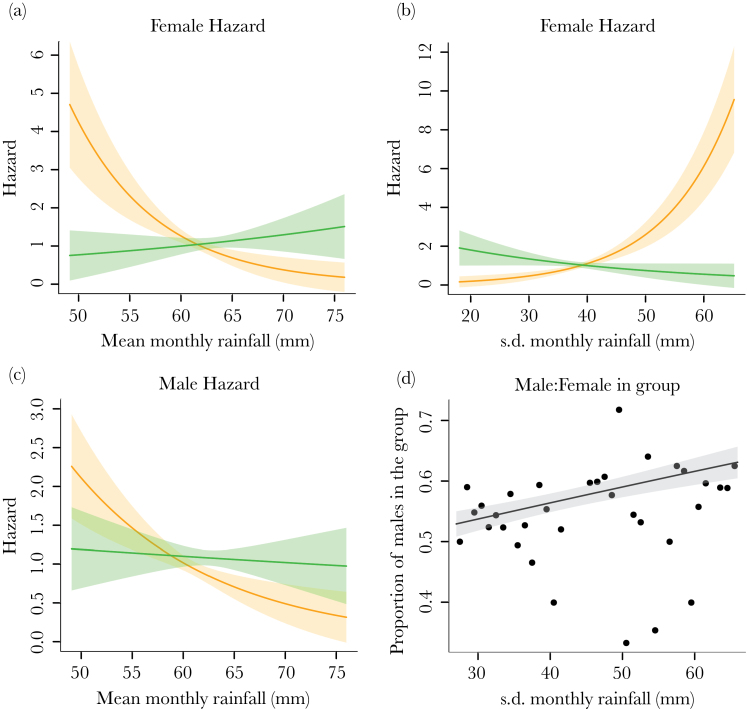
Male and female survival and resulting group sex ratio. Panels (a–c) show how the estimated hazard ratio (± SE) of dying for females (a, b) and males (c) varies with the mean (a, c) and SD (b) of monthly rainfall in the past 12 months, and individuals’ weight gain. The lines show the predictions for the low (orange; 25th percentile: females = −0.85%/day, males = −1.1%/day) and high (green: 75th percentile: females = 2.2%/day, males = 1.8%/day) proportional weight gain rates. Weight gain is categorized here for illustrative purposes and was a continuous variable in our analyses. Panel (d) shows how the proportional of males in a social group at the start of a breeding attempt varies with SD in monthly rainfall in the previous 12 months. The points are the mean values for 1-mm bins, and the line (and shaded area) is the model prediction (± SE).

Mongoose daily weight gains increased with the amount of rainfall in the past 30 days, and female weight changes were more susceptible to this short-term variation in rainfall (female vs. male effect of rainfall: β ± SE = 0.12±0.03, $\chi_{1}^{2}$ = 11.72, *P* ≤ 0.001; Figure 2a). Over a year, this translated into greater increases in weight in both sexes when mean monthly rainfall was higher (mean rainfall: β ± SE = 2.29±0.75, $\chi_{1}^{2}$ = 9.61, *P* = 0.002; Figure 2b). This also appeared to translate into greater annual weight increases in females when rainfall had been more variable (female vs. male effect of SD rainfall: β ± SE = 2.88±1.09, $\chi_{1}^{2}$ = 7.13, *P* = 0.01; Supplementary Table S2). However, our survival analyses (Supplementary Table S3) showed that this effect was due to selective disappearance because females who gained less weight during variable years were at greater risk of dying (female weight change × SD rainfall: β ± SE = −4.03±1.43, $\chi_{1}^{2}$ = 9.13, *P* = 0.002; Figure 3b). The variability of monthly rainfall did not affect male survival, but both males and females who gained less weight were at greater risk of dying in years when mean monthly rainfall had been lower (male weight change × mean rainfall: β ± SE = 2.25±0.74, $\chi_{1}^{2}$ = 7.79, *P* = 0.01; female weight change × mean rainfall: β ± SE = 4.82±2.01, $\chi_{1}^{2}$ = 6.57, *P* = 0.01; Figure 3a,c).

### Social group composition

The adult sex ratio of groups was more male biased when monthly rainfall in the previous 12 months had been more variable (β ± SE = 0.01±0.004, $\chi_{1}^{2}$ = 7.89, *P* = 0.005; Figure 3d), but was not affected by the mean monthly rainfall in the past 12 months ($\chi_{1}^{2}$ = 0.11, *P* = 0.74) or rainfall in the past 30 days ($\chi_{1}^{2}$ = 1.13, *P* = 0.29). The number of adults (potential helpers) in the group was not influenced by the mean or variability of monthly rainfall in the 12 months (mean rainfall: $\chi_{1}^{2}$ = 0.76, *P* = 0.38; SD rainfall: $\chi_{1}^{2}$ = 0.41, *P* = 0.52) or the rainfall in the past 30 days ($\chi_{1}^{2}$ = 2.97, *P* = 0.09).

### Fitness benefits of reproductive and helping effort

**Figure 4:**
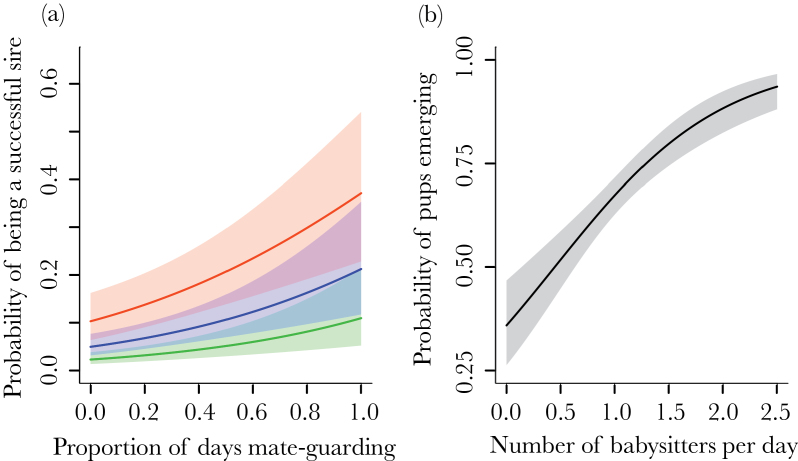
Effect of (a) male mate-guarding effort on their probability siring a pup in the litter and (b) babysitting effort on the probability of a litter emerging from the den. Lines are the predicted relationships from the model (± SE). In (a), the lines show the effect for males of age 3 (green), 5 (blue), and 7 years (orange). Age is categorized here for illustrative purposes and was a continuous variable in our analyses.

Consistent with earlier findings, increased mate-guarding and babysitting behavior had direct and indirect fitness benefits, respectively (Cant 2003; Sanderson, Wang, et al. 2015), but these fitness benefits were not directly influenced by the mean or variability of ecological conditions (Supplementary Table S4). Older males and those investing more in mate-guarding were more likely to sire a pup in a breeding attempt (age: β ± SE = 0.37±0.11, $\chi_{1}^{2}$ = 11.07, *P* ≤ 0.001; mate-guarding: β ± SE = 1.78±0.64, $\chi_{1}^{2}$ = 8.08, *P* = 0.004; Figure 4a; Sanderson, Wang, et al. 2015). The probability of siring a pup was not, however, influenced by the mean or variability of the rainfall in the previous 12 months (mean rainfall: $\chi_{1}^{2}$ = 2.26, *P* = 0.13; SD rainfall: $\chi_{1}^{2}$ = 0.03, *P* = 0.87; Supplementary Table S4). Pups were more likely to emerge from the den (i.e., survive the babysitting period) when there were more babysitters per day (β ± SE = 0.02±0.01, $\chi_{1}^{2}$ = 5.99, *P* = 0.01; Figure 4b; Cant 2003), but this probability of pup emergence was not affected by the mean or variability of the rainfall in the previous 12 months (mean rainfall: $\chi_{1}^{2}$ = 0.36, *P* = 0.55; SD rainfall: $\chi_{1}^{2}$ = 0.59, *P* = 0.44; Supplementary Table S4).

## DISCUSSION

The variability of ecological conditions altered the composition of banded mongoose groups and the patterns of reproductive and cooperative behavior within them. Female mongooses were more sensitive than males to short-term fluctuations in rainfall leading to, in the longer-term, poorer female survival and male-biased group sex ratios when rainfall was more variable. Older males, who have greater access to females and were more likely to sire pups, apparently responded to this reduced number of females in variable conditions by increasing their investment in indirect fitness returns and helping more. In contrast, when mean conditions were better the group sex ratio was unchanged and individuals were in better condition, leading to older males increasing their investment in direct fitness returns by mate-guarding more. These findings demonstrate that individual differences in sensitivity to ecological conditions, and the knock-on effects this has on the composition of animal groups, can influence selection on social behaviors such as cooperative care.

Our findings support suggestions that more variable ecological conditions promote helping behavior (Rubenstein and Lovette 2007; Jetz and Rubenstein 2011; Rubenstein 2011), but do not support the mechanism that Rubenstein (2011) hypothesized produced this pattern. He suggested subordinates were increasing their helping effort in variable conditions as a bet-hedging strategy to reduce their fecundity variance and avoid zero reproductive output. However, our findings show the opposite: helping effort increased in dominant (older) rather than subordinate (younger) males in response to more variable ecological conditions. One explanation for this difference may be that these previous studies have been in birds where individuals tend to choose between 2 strategies to employ for a given breeding period: “help” or “attempt-to-breed” (e.g., Rubenstein 2007; Hatchwell and Sharp 2013). The attempt-to-breed strategy is expected to be riskier, and so involve higher variance in fecundity, for subordinates. As a result, subordinates should be more likely to choose the help strategy in response to factors, such as greater ecological variability, which increase all individuals’ fecundity variance (Rubenstein 2011). In our mongoose system, however, individuals are able to adjust their investment in helping and reproduction over much shorter timescales (given that there are several breeding attempts per year and helping decisions are made on a daily basis) and do not necessarily need to trade off investment in one against the other (see also Sanderson, Stott, et al. 2015). Older males have greater access to mating opportunities with females (Cant 2000; Nichols et al. 2010) and apparently adjusted their helping and reproductive effort according to female availability. Greater ecological variability reduced groups’ relative number of females and led to older males increasing their helping effort. In contrast, improved mean ecological conditions did not change female availability, but did increase male body condition, leading to older males increasing their reproductive effort. Considering differences between social systems in how and when individuals need to decide about their investment in helping and reproduction may, therefore, be important in understanding the impact of ecological conditions on individuals’ relative investment in direct and indirect fitness returns.

Our findings support the hypotheses that older males’ increase in helping behavior in more variable ecological conditions is a response to reduced female availability; however, there are 2 alternative explanations that are also worth considering. First, rather than being a response to a reduced availability of mating opportunities, could this increased helping behavior be compensating for the reduced helping effort available from females? This seems unlikely because the number of adults (potential helpers) in the group was not influenced by ecological conditions and we controlled for the number adults in the group in our model predicting helping behavior. Indeed, helping behavior is male biased in banded mongooses (Cant 2003; Gilchrist and Russell 2007; Hodge 2007), and so the male-biased group compositions in more variable environments implies a larger workforce of available helpers. Second, older males were more likely to sire a pup and increasing babysitting effort improved the chances of at least one pup from a litter surviving the babysitting period. This might suggest that older males’ increased babysitting behavior represents a mechanism for increasing their direct, rather than indirect, fitness returns. However, if this were the case, we would have expected that males’ probability of siring a pup, and so their chance of receiving a direct fitness return from a litter, would also be affected by ecological variability, but it was not. Furthermore, as shown here and previously, in banded mongooses, younger males provide more cooperative care overall than older males (Cant 2003; Gilchrist and Russell 2007; Hodge 2007) and are less likely to have sired a pup in a litter (this study; Sanderson, Wang, et al. 2015). Although increased direct fitness returns may be a side effect of increased helping behavior by older males, our results suggest that indirect fitness returns are a major driver of variation on helping behavior in banded mongooses.

Our study provides empirical evidence that helping in older males is promoted in more variable environments due to sex differences in sensitivity to ecological conditions, and the knock-on effect this has on social group composition. Recent theory has shown that where sex differences in survival exist, the sex with better survival should experience greater selection for a particular trait (Mullon et al. 2014). Our findings support this prediction in the context of cooperative care, showing a pattern consistent with greater selection for helping in males when increased ecological variability reduces female survival. Additionally, Mullon et al.’s (2014) model predicts that 1) the sex with better survival will have lower reproductive variance and 2) selection should favor behaviors in this sex that reduce the higher reproductive variance in the other sex. Our study does not test the first of these predictions but does support the second, showing increased helping in more variable environments by the sex (males) with better survival. Whether female reproductive variance increases with variance in ecological conditions and whether males’ helping effort reduces this variance is unclear; however, helping has been shown to reduce reproductive variance in superb starlings (Rubenstein 2011). Our results, therefore, raise the intriguing possibility that differences between sexes (or any phenotypes) in their survival under certain ecological conditions act to promote and maintain helping behavior and that this selection on helping comes about through changes in social group composition.

Sex differences in sensitivity to ecological conditions have been shown in many mammal species (e.g., Conradt et al. 2000; Coulson et al. 2001; Bowyer 2004). These differences are often attributed to sexual dimorphism leading to greater sensitivity in the larger sex, usually males, due to their greater energetic requirements (Isaac 2005), but can also be the result of increased energetic demands of pregnancy and lactation in females (Key and Ross 1999; Main 2008). Banded mongooses show relatively low sexual dimorphism suggesting that the greater environmental sensitivity we find in female mongooses is due to the energetic requirements of reproduction. Female mongooses are pregnant for an average of about 30% of each year (Marshall HH et al., unpublished data), which may impair their ability to respond to changes in ecological conditions as quickly as males. This inability to respond to ecological changes may be because, compared with males, female reproduction is under stronger social control through eviction and infanticide by other females (Cant et al. 2010, 2014). Indeed, where ecological conditions have been found to affect female reproduction it was only in breeding attempts where an eviction occurred (Nichols, Bell, et al. 2012). Eviction of females may also provide an alternate explanation for our sex ratio result because Nichols, Bell, et al. (2012) suggested rainfall affected the probability of an eviction occurring; however, a more recent comprehensive analysis (using data on 47 rather than 8 eviction events) found that rainfall did not predict the likelihood of an eviction (Faye J. Thompson et al., in preparation). Overall, our results are consistent with previous suggestions that helping in banded mongooses imposes longer-term costs on future reproductive potential in females (Hodge 2007; Bell 2010).

Our results show how increased ecological variability can, in the short term, promote helping behaviors through effects on survival and group composition. However, in the longer-term sustained levels of greater ecological variability (e.g., such as those predicted under climate change, IPCC 2013) are likely to produce negative effects on individual and group fitness, for example, because increasingly male-biased sex ratios make mating opportunities with females harder to find, leading to Allee-like inverse density-dependent effects (Courchamp et al. 1999). It is worth noting, however, that tests of the effect of ecological variability on helping behavior have been limited to tropical systems (Rubenstein and Lovette 2007; Rubenstein 2011; Gonzalez et al. 2013; but see Jetz and Rubenstein 2011 for a global analysis) and so tests in temperate systems, with different patterns of seasonality, would be valuable. Nonetheless, these findings support the broad idea that individual differences in behavioral responses to environmental change can have an important role in ecology, evolution, and conservation (Sih et al. 2010; Dall et al. 2012; Pelletier and Garant 2012; Wong and Candolin 2015), particularly in social species whose complex interindividual interactions can lead to unexpected effects (Blumstein 2012; Wong 2012). They also support recent models suggesting that changes in ecological variability may also play as important a role as changes in mean conditions in evolutionary and population processes (Botero et al. 2014; Lawson et al. 2015). A greater understanding of the effects of ecological variability on individual survival and behavior is likely to be important in predicting the impacts of environmental change, particularly because climate change is expected to increase environmental variability as well as changing its average (IPCC 2013). This is further emphasized by recent studies suggesting that sex differences in sensitivity to ecological conditions may have an important influence on the population-level impacts of climate change (Marchand et al. 2014; Berger et al. 2015).

In conclusion, our study provides the first empirical evidence for increased individual helping effort in more variable ecological conditions and for a mechanism underpinning this effect. Poorer female survival in more variable conditions appears to result in older males helping more due to the lack of mating opportunities. These results suggest that individual differences in susceptibility to ecological variability, and the consequences of this for social group composition, can have an important influence on selection for cooperative care and other social traits.

## SUPPLEMENTARY MATERIAL

Supplementary material can be found at http://www.beheco.oxfordjournals.org/

## FUNDING

The research was funded by a European Research Council Starting Grant (SOCODEV) and a Natural Environment Research Council (UK) Standard Grant (NE/J010278/1).

## Supplementary Material

Supplementary Data
