# Supplementary material for: Variable ecological conditions promote male helping by changing banded mongoose group composition
Source: Behav Ecol. 2016 Jan 26;27(4):978–87. doi: 10.1093/beheco/arw006 (PMC4943108; doi:10.1093/beheco/arw006)
Supplement: Supplementary Data [file supp_27_4_978__index.html]

Variable ecological conditions promote male helping by changing banded mongoose group composition — Variable ecological conditions promote male helping by changing banded mongoose group composition — Variable ecological conditions promote male helping by changing banded mongoose group composition — Variable ecological conditions promote male helping by changing banded mongoose group composition — Supplementary Data 

# Variable ecological conditions promote male helping by changing banded mongoose group composition

## Supplementary Data

Data files

- Supplementary Data - Supplementary Data
