# Supplementary material for: Variable ecological conditions promote male helping by changing banded mongoose group composition
Source: Behav Ecol. 2016 Jan 26;27(4):978–87. doi: 10.1093/beheco/arw006 (PMC4943108; doi:10.1093/beheco/arw006)
Supplement: Supplementary Data [file supp_arw006_Online_supporting_information_revised_final.pdf]

Supporting Information for Marshall et al. submitted to *Behavioral Ecology*

Table S1: Models predicting reproductive and helping effort in males and females. Significant effects involving the mean and variability of ecological conditions are in bold. All models were fitted with mongoose, breeding attempt and social group ID as random intercepts using a binomial error structure and logit link function.

	Response	Fixed effect	β	s.e.	χ^2	p
Males	Mate-guarding effort	intercept	-8.36	5.12		
		age	1.04	0.93		
		mean rainfall in previous 12 months	-0.09	0.05		
		s.d. of rainfall in previous 12 months	-0.01	0.01	0.66	0.41
		mean rainfall in first year	0.09	0.07		
		s.d. rainfall in first year	0.07	0.03		
		male weight	0.00	0.00	3.88	0.05
		overlap with babysitting period	-0.45	0.24	3.60	0.06
		overlap with escorting period	0.22	0.34	0.41	0.52
		ratio of males to females	-0.27	0.10	11.53	< 0.001
		**age x**				
		**mean rainfall in previous 12 months**	**0.02**	**0.01**	**5.99**	**0.01**
		s.d. of rainfall in previous 12 months	-0.01	0.01	1.72	0.19
		mean rainfall in first year	-0.03	0.01	7.10	0.01
		s.d. rainfall in first year	-0.01	0.01	5.79	0.02
	Babysitting effort	intercept	0.74	1.70		
		age	-0.62	0.17		
		mean rainfall in previous 12 months	-0.02	0.02	0.97	0.32
		s.d. of rainfall in previous 12 months	-0.01	0.01		
		mean rainfall in first year	-0.01	0.01	0.96	0.33
		s.d. rainfall in first year	-0.04	0.02	4.74	0.03
		male weight	0.002	0.001	6.90	0.01
		overlap with oestrus period	-0.22	0.12	2.97	0.08
		number of adults in group	-0.04	0.01	11.09	<0.001
		**age x**				
		mean rainfall in previous 12 months	0.001	0.01	0.01	0.92

		**s.d. of rainfall in previous 12 months**	**0.01**	**0.004**	**5.34**	**0.02**
		mean rainfall in first year	-0.003	0.01	0.05	0.83
		s.d. rainfall in first year	0.002	0.004	0.12	0.72
Females	Probability of being in oestrus	intercept	0.81	4.47		
		age	0.11	0.20	0.29	0.59
		mean rainfall in previous 12 months	-0.05	0.04	2.16	0.14
		s.d. of rainfall in previous 12 months	0.01	0.03	0.06	0.80
		mean rainfall in first year	0.01	0.06	0.02	0.89
		s.d. rainfall in first year	0.03	0.02	2.59	0.11
		female weight	0.002	0.002	1.12	0.29
		overlap with babysitting period	-0.68	0.58	1.41	0.23
		overlap with escorting period	1.94	1.21	3.35	0.07
		number of females in group	-0.37	0.12	10.36	0.00
		age x				
		mean rainfall in previous 12 months	-0.03	0.02	1.30	0.25
		s.d. of rainfall in previous 12 months	0.03	0.02	3.01	0.08
		mean rainfall in first year	0.02	0.05	0.19	0.67
		s.d. rainfall in first year	0.03	0.02	1.83	0.18
	Probability of getting pregnant	intercept	1.79	4.96		
		age	0.08	0.19	0.09	0.77
		mean rainfall in previous 12 months	-0.06	0.04	1.97	0.16
		s.d. of rainfall in previous 12 months	-0.01	0.03	0.10	0.76
		mean rainfall in first year	0.01	0.06	-0.02	1.00
		s.d. rainfall in first year	0.01	0.02	0.02	0.89
		female weight	0.003	0.002	1.84	0.18
		overlap with babysitting period	-0.01	0.59	-0.04	1.00
		overlap with escorting period	1.16	1.19	1.15	0.28
		number of females in group	-0.27	0.13	6.22	0.01
		age x				
		mean rainfall in previous 12 months	-0.02	0.02	0.72	0.40
		s.d. of rainfall in previous 12 months	0.03	0.02	1.61	0.20
		mean rainfall in first year	0.03	0.05	0.49	0.49

	s.d. rainfall in first year	0.01	0.02	0.54	0.46
Babysitting effort	intercept	-5.55	3.69		
	age	1.17	0.96		
	mean rainfall in previous 12 months	-0.02	0.01	2.53	0.11
	s.d. of rainfall in previous 12 months	0.00	0.01	0.11	0.74
	mean rainfall in first year	0.10	0.06		
	s.d. rainfall in first year	-0.04	0.02		
	male weight	0.0001	0.0005	0.02	0.90
	overlap with oestrus period	0.13	0.15	0.79	0.37
	number of adults in group	-0.004	0.01	0.09	0.77
	age x				
	mean rainfall in previous 12 months	-0.01	0.01	0.52	0.47
	s.d. of rainfall in previous 12 months	0.005	0.01	0.57	0.45
	mean rainfall in first year	-0.03	0.02	4.08	0.04
	s.d. rainfall in first year	0.01	0.01	7.07	0.01

Table S2: Models predicting changes in daily and annual weight. Significant effects involving the mean and variability of ecological conditions are in bold. [a] reference level = males. Both models were fitted with mongoose and social group ID as random intercepts using a normal error structure.

Response	Fixed effect	β	s.e.	χ^2	p
Daily weight change	intercept	198.02	11.94		
	age	-5.49	4.01	20.41	< 0.001
	sex[a]	2.43	0.54		
	rainfall in past 30 days	0.05	0.02		
	morning weight	-0.11	0.01	160.61	< 0.001
	age x rainfall in past 30 days	0.01	0.01	0.23	0.63
	**sex[a] x rainfall in past 30 days**	**0.12**	**0.03**	**11.72**	**< 0.001**
Annual weight change	intercept	466.13	111.73		
	age	-10.17	4.23	6.00	0.01
	sex[a]	-157.51	44.56		
	**mean rainfall in previous 12 months**	**2.29**	**0.75**	**9.61**	**0.002**
	s.d. of rainfall in previous 12 months	0.03	0.62		
	mean rainfall in first year	-1.23	1.05	1.38	0.24
	s.d. rainfall in first year	0.07	0.46	0.02	0.90
	weight at the start of the year	-0.32	0.05	40.48	< 0.001
	age x				
	mean rainfall in previous 12 months	-0.48	0.48	1.05	0.31
	s.d. of rainfall in previous 12 months	-0.03	0.39	0.02	0.90
	mean rainfall in first year	0.19	0.72	0.12	0.73
	s.d. rainfall in first year	-0.06	0.34	0.03	0.86
	**sex[a] x**				
	mean rainfall in previous 12 months	0.04	1.53	0.00	0.98
	**s.d. of rainfall in previous 12 months**	**2.88**	**1.09**	**7.13**	**0.01**
	mean rainfall in first year	1.37	2.36	0.34	0.56
	s.d. rainfall in first year	-0.63	0.94	0.46	0.50

Table S3: Cox proportional hazard models predicting the hazard of death in a given year in females and males. Significant effects involving the mean and variability of ecological conditions are in bold. Both models included social group ID as a frailty term.

Response	Fixed effect	β	s.e.	χ^2	p
Female Hazard	proportional weight change	-144.12	111.23		
	mean rainfall in previous 12 months	-0.08	0.05		
	s.d. of rainfall in previous 12 months	0.06	0.03		
	mean rainfall in first year	0.05	0.09	0.32	0.58
	s.d. rainfall in first year	0.03	0.02	1.34	0.24
	**proportional weight change x**				
	**mean rainfall in previous 12 months**	**4.82**	**2.01**	**6.57**	**0.01**
	**s.d. of rainfall in previous 12 months**	**-4.03**	**1.43**	**9.13**	**0.002**
Male Hazard	proportional weight change	-132.10	43.59		
	mean rainfall in previous 12 months	-0.05	0.04		
	s.d. of rainfall in previous 12 months	-0.02	0.02	0.86	0.35
	mean rainfall in first year	0.03	0.04	0.52	0.46
	s.d. rainfall in first year	-0.01	0.02	0.22	0.64
	**proportional weight change x**				
	**mean rainfall in previous 12 months**	**2.25**	**0.74**	**7.79**	**0.01**
	s.d. of rainfall in previous 12 months	-0.30	0.64	0.33	0.55

Table S4: Models predicting the direct and indirect fitness benefits of mate-guarding and babysitting behaviour, respectively. Significant behavioural effects are shown in bold. The probability of successfully siring a pup model was fitted with mongoose, breeding attempt and social group ID as random intercepts. The probability of pups emerging model was fitted with social group ID as a random intercept. Both models were fitted using a binomial error structure and logit link function.

Response	Fixed effect	β	s.e.	χ^2	p
Probability of successfully siring a pup	intercept	-2.35	2.56		
	age	0.37	0.11	11.07	< 0.001
	**mate-guarding effort**	**1.78**	**0.64**	**8.08**	**0.004**
	mean rainfall in previous 12 months	-0.06	0.04	2.26	0.13
	s.d. of rainfall in previous 12 months	-0.002	0.02	0.03	0.87
	rainfall in past 30 days	0.02	0.01	10.80	0.001
	ratio of males to females	0.13	0.16	0.87	0.35
	age x				
	mean rainfall in previous 12 months	-0.03	0.02	2.97	0.08
	s.d. of rainfall in previous 12 months	0.0005	0.01	0.11	0.74
	rainfall in past 30 days	-0.001	0.003	0.57	0.45
	mate-guarding effort x				
	mean rainfall in previous 12 months	-0.02	0.12	-0.07	1.00
	s.d. of rainfall in previous 12 months	0.01	0.07	0.05	0.83
	rainfall in past 30 days	0.03	0.02	3.28	0.07
Probability of pups emerging	intercept	-1.36	1.91		
	**mean number of babysitters per day**	**1.30**	**0.42**	**11.72**	**< 0.001**
	mean rainfall in previous 12 months	0.02	0.03	0.36	0.55
	s.d. of rainfall in previous 12 months	-0.01	0.02	0.59	0.44
	rainfall in past 30 days	0.01	0.01	1.45	0.23
	number of females giving birth	0.04	0.11	0.13	0.72
	number of adults in the group	-0.03	0.04	0.55	0.46
	mean number of babysitters per day x				
	mean rainfall in previous 12 months	-0.03	0.06	0.99	0.32
	s.d. of rainfall in previous 12 months	-0.001	0.04	0.26	0.61
	rainfall in past 30 days	-0.01	0.01	0.70	0.40
	number of females giving birth	-0.25	0.22	0.88	0.35
	number of adults in the group	0.08	0.07	0.46	0.50
